# Outcomes of Propeller Flap in Eyelid Reconstruction

**DOI:** 10.7759/cureus.14509

**Published:** 2021-04-15

**Authors:** Nausheen Hayat, Saad Jan, Saad Aslam, Muhammad Sohaib Asghar

**Affiliations:** 1 Ophthalmology, Jinnah Postgraduate Medical Centre, Karachi, PAK; 2 Ophthalmology, Dow University of Health Sciences, Karachi, PAK; 3 Internal Medicine, Dow University of Health Sciences, Karachi, PAK

**Keywords:** eyelid tumors, reconstruction, basal cell carcinoma of lids, flap, skin cancers

## Abstract

The purpose of this case report is to demonstrate the outcomes of reconstruction in small-to-moderate-sized benign tumors of eyelids by using the propeller flap technique. Propeller flaps have been used by plastic surgeons for facial reconstruction and other body parts. However, very few oculoplastic surgeons have utilized this technique in eyelid reconstructive surgeries. We have substantiated this technique and its outcomes in this case report. It is a case series consisted of two patients reporting in the orbit-oculoplastics clinic with suspicious basal cell carcinoma of eyelids, covering less than half of the eyelid. Both the patients underwent tumor excision and reconstruction, with a cutaneous propeller flap supplied by a pedicle. Both the patients recovered well and without any complication. The specimens removed from both the patients were sent for histopathology and the biopsy results revealed both lesions were margin-free basal cell carcinoma. The reconstruction of eyelid defects is challenging due to the small area and cosmetic appearance. Propeller flaps are a reasonable choice of treatment in small to moderately large-sized defects with minimal complications, yet better cosmetic appearance.

## Introduction

Introduced in 1991 by Hayakusoku et al. [1] and further expanded by Hallock and Teo in 2006 [2,3], propeller flaps are defined as an “island flap that reaches the recipient site through an axial rotation.” This definition was agreed upon in the first Tokyo meeting on perforator and propeller flaps [4]. Another important outcome of the Tokyo meeting was the establishment of a classification system for propeller flaps, which included naming different propeller flaps in accordance with their nourishing vessels. The classification system is based on the nourishing pedicle (subcutaneous pedicled propeller flap, perforator pedicled propeller flap, and supercharged propeller flap); the degrees of skin island rotation (90 to 180 degrees); axis type (central or acentric); and where possible, the artery of origin of the perforator.

In recent years, propeller flaps have enjoyed a steadily increasing popularity and the technique has been improved and further elaborated by several authors [5-10]. These flaps are mostly used in plastic and reconstructive surgery elsewhere in the body and have been seldom reported in orbital or periorbital reconstruction in our region. However, Rajak et al. in their study have demonstrated its successful use in eight patients with nasolabial propeller flap [11]. Therefore, in our case series, we present the utilization of propeller flaps as a viable technique to cover small to moderately large periocular defects. To the best of our knowledge, no local work has been done in this regard.

## Case presentation

In this prospective case series, two patients reported through the outpatient department and were referred to the orbit-oculoplastic clinic of Jinnah Postgraduate Medical Centre (JPMC). Both the patients who were operated on between June 2019 and October 2019 were thoroughly evaluated. A comprehensive history and relevant ophthalmic examinations were performed. Informed consent was taken and explained to both the patients. Among these two patients, one was male and the other was female. All surgeries were performed by the same surgeon under local infiltrating anesthesia. The inclusion criteria consisted of patients presenting with lesions between the size of three to four centimeters (cm) and apparently benign in nature, whereas recurrent, large-sized, fast-growing, or aggressive looking lesions patients with any underlying systemic or skin disease were excluded.

**Figure 1:**
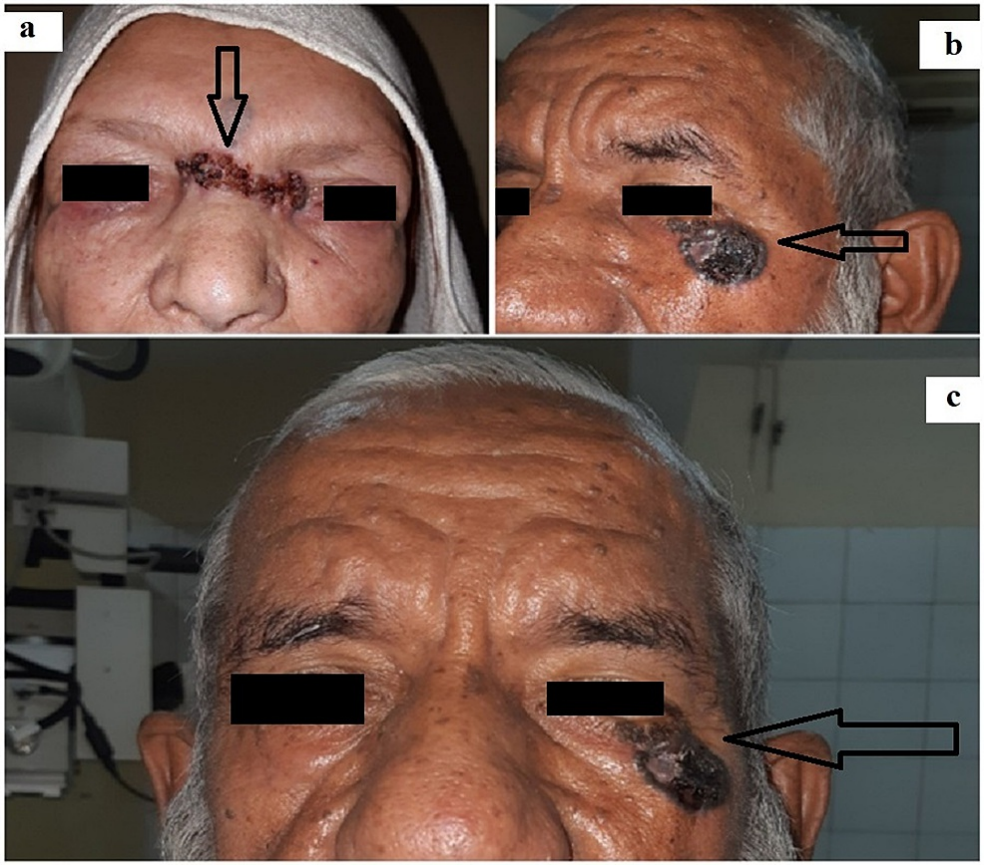
Frontal view of female patient with a lesion of 3.5 cm × 1.5 cm (a), lateral view of male patient with a lesion of 3 cm × 2 cm (b), frontal view of male patient with lesion over left lower lid (c).

The first patient was a 58-year-old female with no known comorbidities, who presented with a lesion measuring approximately three and a half cm horizontally and one and a half cm vertically (3.5 cm × 1.5 cm), extending from the left medial canthus to the nasal bridge. The second patient was a 62-year-old male with no known comorbidities, who presented with a blackish lesion involving lateral one-third of the left lower lid and measured approximately three by two cm (3 cm × 2 cm) as shown in Figures 1A-1C.

**Figure 2:**
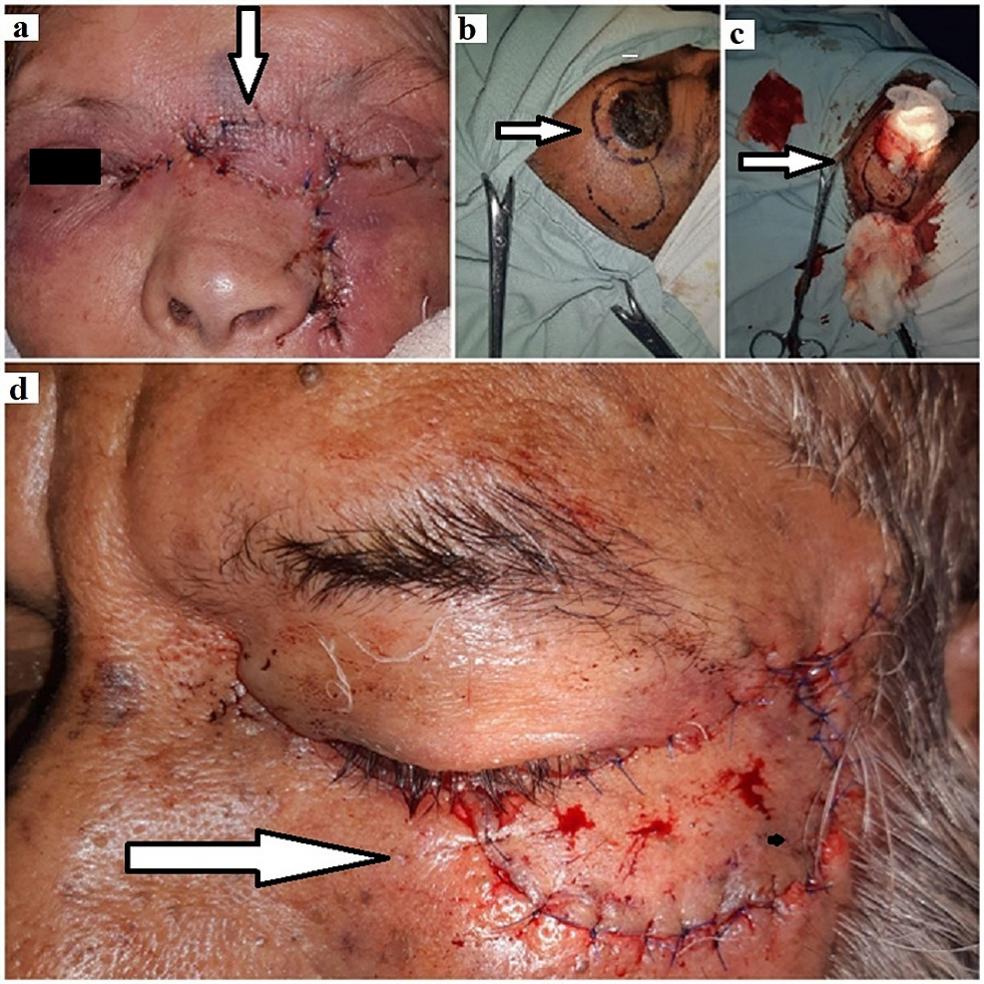
Nasolabial flap of female patient (a), procedure of flap dissection (b, c), and zygomatic flap of male patient (d).

After taking informed consent, the patients were evaluated and prepared for surgery. A single-staged surgery with excision and reconstruction was planned. The surgery was performed under local infiltrating anesthesia (2% lidocaine with epinephrine at 1:200,000). Firstly, we marked the boundaries of the malignant tissue for wide excision with a sufficient four millimeters (mm) lesion-free margin and then carefully excised along with the markings. In the first case, after taking the template from the defect, a slightly larger sized left nasolabial flap was designed from the orbicularis muscle, with its acentric pedicle at the upper end near the lesion, while the tail of the flap was at the lower end, which was rotated 90 degrees on its axis to cover the defect. Whereas in case two, a zygomatic flap was recruited adjacent to the lesion, laterally, with its acentric pedicle near the defect to rotate at 90 degrees, as shown in Figures 2A-2D.

Flaps were then raised with careful dissection with the help of cautery in the subcutaneous or suborbicularis plane as per the desired flap thickness. The location of the pedicle is determined by the site of the defect and the positioning of the flap but is typically one-quarter of the flap diameter away from the defect. The flap can be thinned, but not excessively in order to preserve the subdermal plexus and to maintain flap vascularity. A wide pedicle is created initially and it is gradually reduced whilst checking the rotation after each reduction until the pedicle has reached the maximum diameter that will allow adequate rotation of the flap without excessive torque. Typically, a pedicle of diameter 6-10 mm and length of 10-14 mm is created. The donor site and flap are then sutured into position with prolene suture (size 6-0).

**Figure 3:**
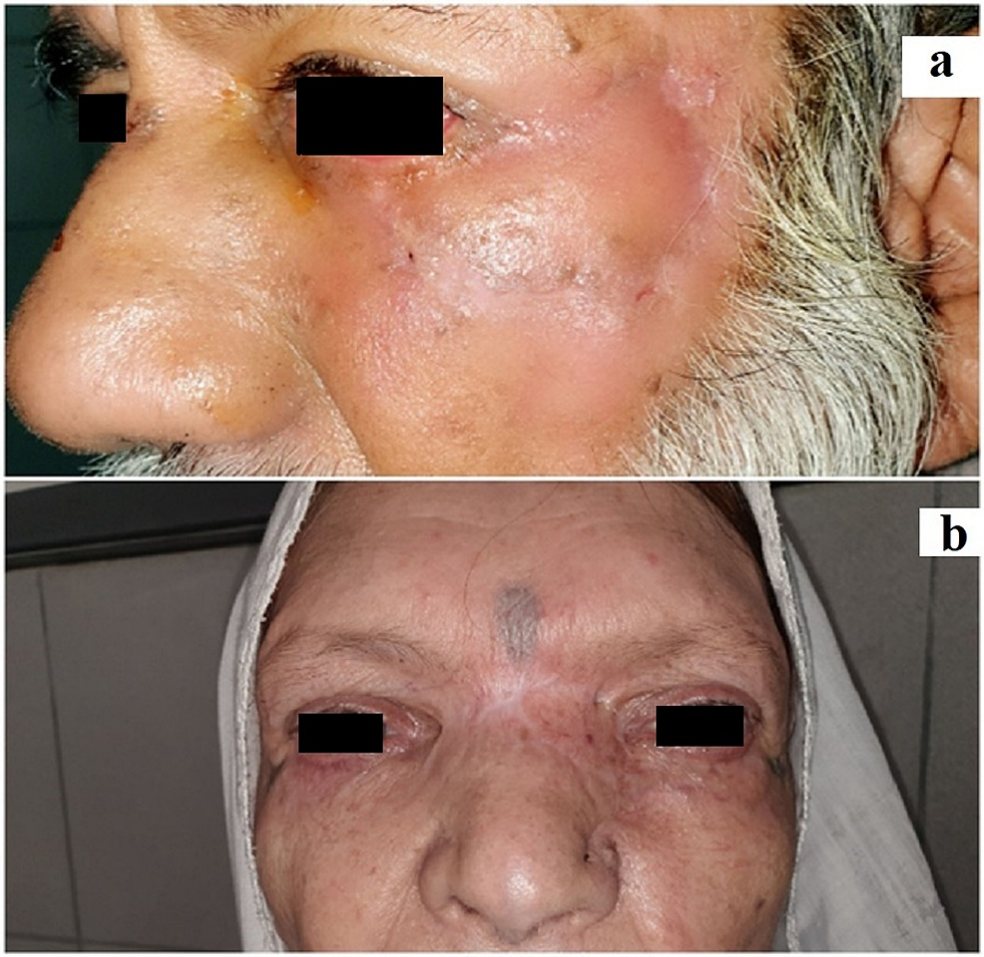
Lateral view of operative site of male patient (a) and postoperative frontal view of female patient (b).

Postoperatively, systemic and local antibiotics with additional vitamin C supplements were given and both the donor and recipient site were examined regularly for any complications. Cold sponging was also advised to reduce edema. Sutures were removed after one week as shown in Figures 3A, 3B.

Both patients underwent surgery to remove lesions from periorbital areas and the resultant skin and soft tissue defect were then covered with a propeller flap. There were no intra-operative complications. In both the patients, marked localized edema was observed on the first post-operative day, which was resolved within two weeks. The specimens removed from both the patients were sent for histopathology after properly marking and labeling them. The biopsy results revealed both lesions were margin-free basal cell carcinoma. The patients were followed for four months post-operatively and both patients healed well without any areas of necrosis with excellent cosmetic and functional outcomes. Here, it may be worth mentioning that in the first case trapdoor effect was observed; however, spontaneous resolution occurred within two months. In the end, both patients were satisfied with their cosmetic and functional outcomes and were potentially cured of basal cell carcinoma as well.

## Discussion

Although popular in reconstructive surgery, propeller flaps are seldom reported for the reconstruction of the periocular area in our region. The literature was scarce in this area a few years back, despite the fact that they have proven to be extremely favorable for extensive reconstructions, keeping in view the scarcity of the immediately neighboring skin [11]. Rotation in propeller flaps is more flexible as there are no limitations of the length-width ratio due to the profuse blood supply of the facial region [12].

Otherwise problematic, the propeller flap has made transposition of sizeable flaps over growing distances relatively easy when compared with skin pedicle flaps, and that too without any contortion at the base of the flap. This, in the periocular region, is of paramount importance as there is already an insufficient amount of skin in the immediate vicinity but there is ample redundant skin in the adjoining areas like the perinasal area and the temples [11]. There have been several other studies that have successfully implemented the propeller flap technique in closing large orbital defects following extensive tumor resections in the periorbital region. Rajak et al. [11] used propeller flaps in multiple periocular defects and were successful in closing the defects following lid tumor resection surgeries, with only one patient developing trapdoor effect, which led to a revision surgery correcting the defect. Ding et al. [12] also successfully utilized this technique and closed a large lower eyelid defect that resulted due to extensive tumor resection. They used a one-stage reconstruction approach, similar to ours. Another study by Baltu et al. [13] showed propeller flaps successfully used in single-stage reconstruction procedures, used to cover large periocular defects in a variety of patients with generally favorable outcomes.

Propeller flaps are advantageous to other techniques in closing a periocular defect because they provide the best possible aesthetic outcome as they have excellent resemblance in color and texture because the donor area is in close proximity with the defect and the scars are not very prominent. Also, the chances of ectropion and lower eyelid retractions are minimal [14]. There are some expected complications of propeller flaps, which include wound or flap dehiscence, hematoma formation, flap necrosis, wound infection and edema. Another complication which one must expect is the trapdoor effect, which basically is the elevation or bulging of tissues within the boundaries of scar [15]. This complication was seen in one of our patients and occurred due to scar hypertrophy and flap base contraction, which is also augmented by venous and lymphatic outflow impedance. Usually, it resolves spontaneously though it may occasionally require steroid injections, or in some rare cases, revision surgery.

## Conclusions

Propeller flaps have significantly improved the aesthetic and functional outcomes in cases of periocular reconstructive surgery because of their origin in the vicinity of the periocular defect, which leads to their harvesting with relative ease on the surgeon’s part and better overall outcomes. This study, however, had a few limitations, such as small data size and small-sized lesions. It is recommended to carry out this trial in a large number of patients with a variety of defects or diseases to obtain a significant result.

## References

[1] Hyakusoku H, Yamamoto T, Fumiiri M (1991). The propeller flap method. Br J Plast Surg.

[2] Hallock GG (2006). The propeller flap version of the adductor muscle perforator flap for coverage of ischial or trochanteric pressure sores. Ann Plast Surg.

[3] Teo TC (2006). Perforator local flaps in lower limb reconstruction. Cirugia Plastica Ibero-Latinoamericana.

[4] Pignatti M, Ogawa R, Hallock GG (2011). The "Tokyo" consensus on propeller flaps. Plast Reconstr Surg.

[5] Pignatti M, D'Arpa S, Cubison TC (2009). Novel fasciocutaneous flaps for the reconstruction of complicated lower extremity wounds. Tech Orthop.

[6] D'Arpa S, Cordova A, Pirrello R, Moschella F (2009). Free style facial artery perforator flap for one stage reconstruction of the nasal ala. J Plast Reconstr Aesthet Surg.

[7] D'Arpa S, Cordova A, Pignatti M, Moschella F (2011). Freestyle pedicled perforator flaps: safety, prevention of complications, and management based on 85 consecutive cases. Plast Reconstr Surg.

[8] Hamdi M, Van Landuyt K, Monstrey S, Blondeel P (2004). Pedicled perforator flaps in breast reconstruction: a new concept. Br J Plast Surg.

[9] Lecours C, Saint-Cyr M, Wong C, Bernier C, Mailhot E, Tardif M, Chollet A (2010). Freestyle pedicle perforator flaps: clinical results and vascular anatomy. Plast Reconstr Surg.

[10] Mateev MA, Kuokkanen HO (2012). Reconstruction of soft tissue defects in the extremities with a pedicled perforator flap: series of 25 patients. J Plast Surg Hand Surg.

[11] Rajak SN, Huilgol SC, Murakami M, Selva D (2018). Propeller flaps in eyelid reconstruction. Eye (Lond).

[12] Ding JP, Chen B, Yao J (2018). Lateral orbital propeller flap technique for reconstruction of the lower eyelid defect. Ann R Coll Surg Engl.

[13] Baltu Y, Uzun H, Dölen UC, Özyurtlu M (2016). Central artery perforator propeller flap for nasal and medial canthal defects. J Plast Reconstr Aesthet Surg.

[14] Uslu A (2019). Use of a perforator/subcutaneous pedicled propeller flap for reconstruction of lower eyelid defects. J Plast Reconstr Aesthet Surg.

[15] Koranda FC, Webster RC (1985). Trapdoor effect in nasolabial flaps. Causes and corrections. Arch Otolaryngol.

